# Deaths of female sex workers and sexually exploited adolescent girls during the COVID-19 pandemic in Kenya, Nigeria, and the Democratic Republic of the Congo: Results of a cross-sectional study

**DOI:** 10.1371/journal.pgph.0005781

**Published:** 2026-08-12

**Authors:** Brian Willis, Isabella Danel, Regan Moss, Emily Perttu, Heather Thompson, Swarna Weerasinghe, Grace Kamau, Wendy L. Macias-Konstantopoulos

**Affiliations:** 1 Global Health Promise, Portland, Oregon, United States of America; 2 Adjunct Assistant Professor, Community Health Sciences, School of Public Health, University of Illinois, Chicago, Illinois, United States of America; 3 McMaster Faculty of Medicine, Hamilton, Ontario, Canada; 4 Department of Community Health and Epidemiology, Dalhousie University, Faculty of Medicine, Halifax, Nova Scotia, Canada; 5 African Sex Workers Alliance, Africa; 6 Center for Social Justice and Health Equity, Department of Emergency Medicine, Massachusetts General Hospital and Harvard Medical School, Boston, Massachusetts, United States of America; University of Ghana, GHANA

## Abstract

Deaths among sexually exploited adolescent girls (SEAG) and female sex workers (FSW) are under-researched and under-reported. Although excess mortality during the COVID-19 pandemic has been documented among vulnerable populations, specific data on SEAG and FSW remain scarce. This lack of reliable data limits the ability of sex worker organizations and public health programs to design effective interventions or respond to shifting risks. We applied the Community Knowledge Approach to collect peer-reported data on deaths of FSW. Participants included 853 FSW across eight cities in Kenya, Nigeria, and the Democratic Republic of the Congo (DRC). Data collection took place in 2022 (Kenya and Nigeria) and 2023 (DRC), covering deaths during the COVID-19 pandemic. A total of 3,810 FSW deaths were reported, including 324 SEAG (<18 years) deaths: 1,482 deaths in the DRC, 870 in Kenya, and 1,458 in Nigeria. Overall, maternal causes resulted in 28.5% of deaths, with abortion alone responsible for 19.2%. Homicide was the leading individual cause (22.6%), followed by HIV/AIDS (11.7%) and suicide (8.8%). Of 280 peripartum deaths, 175 resulted in stillbirths or neonatal deaths. The majority of the deceased (2,243; 58.9%) were mothers, leaving behind 4,310 children. Among deaths of SEAG, maternal causes and homicide were the leading causes of death. Over one-third (110; 35.0%) were pregnant at the time of death; another third (105; 33.5%) were already mothers. The youngest reported age at death was 11. In the DRC, 30.0% of deceased SEAG were daughters of FSWs. This study reveals a substantial, largely invisible death toll among SEAG and FSW during the pandemic. Many of these deaths could have been prevented through existing, cost-effective interventions. The findings underscore an urgent need for targeted public health responses and mortality surveillance for this neglected population—both to prevent further deaths and to mitigate the long-term consequences for the children left behind.

## Introduction

Female sex workers (FSW) in low-and-middle-income countries (LMIC), many of whom are mothers, are a highly marginalized group of women [1]. Due to their social situation and economic precarity, many FSW experience elevated risks for a range of poor health and social outcomes, including HIV and other sexually transmitted infections, gender-based violence, mental health problems ranging from depression to suicidality, and substance use [2]. In addition to these vulnerabilities, FSW often experience heightened barriers to healthcare and support services, such as unaffordable costs, concerns about confidentiality, stigma related to sex work, and disrespectful treatment from providers [3,4].

These structural barriers and cumulative risks likely increase the risk of premature mortality for FSW. However, there is limited information on the causes of death among FSW, particularly in LMIC. A 2019 study identified maternal causes, particularly abortion-related complications, murder, HIV/AIDS, and suicide, as leading causes of death among FSW in LMIC [2]. Identifying the leading causes of death among FSW is critical to designing preventive policies and programs, monitoring their effectiveness, and detecting shifts in causes of death trends that would necessitate modifications or additional interventions.

During the COVID-19 pandemic, globally there was excess mortality and shifts in the leading causes of death [5,6]. The World Health Organization (WHO) estimated that COVID-19 resulted in approximately 14.9 million excess deaths from the COVID-19 pandemic [7–9]. While many deaths were directly caused by the COVID-19 virus, a large number of other deaths resulted from the social and economic impact of the pandemic [10]. These impacts were particularly severe in LMIC where the pandemic further strained already austere social and economic conditions [5].

Many factors, such as disruption to healthcare systems, contributed to excess mortality during the pandemic [11–16]. Lockdowns impeded travel to medical care and medical services, including maternal healthcare, especially in LMIC [11]. In addition, studies from LMIC document interruptions in maternal health services during the pandemic, disruptions in access to contraception, and a decrease in access to safe abortion and post-abortion care [12]. Lockdowns also resulted in lost income to some workers, including FSW, which increased the difficulties pregnant women experienced accessing and affording healthcare and HIV care in sub-Saharan Africa [13–15]. Indeed, excess deaths due to a lack of health services were estimated to be 2.7 times higher than the number of deaths directly attributable to COVID-19 infections [16].

The pandemic also had a disproportionate impact on vulnerable populations, which includes FSW [17]. Socioeconomic factors, including limited education, poverty, poor housing, low income, and living in overcrowded households, were also risk factors for adverse outcomes from infection with COVID-19 [18]. These risk factors are common among FSW in LMIC [14]. The COVID-19 pandemic also led to increased work-related victimization, substance use, police violence against FSW, and food insecurity [19]. Finally, lockdowns in some countries increased economic precarity among FSW [15].

During the COVID-19 pandemic, studies documented increased violence against women. However, data on FSW were not included in a global study of violence against women during this period [20]. Similarly, country-level studies, including from Kenya and Nigeria, reported an increase in gender-based violence (GBV), femicide, and interpersonal violence (IPV), during the pandemic but did not report data specific to FSW [21–23]. Although violence against FSW, including homicides, is well documented, no study assessed whether mortality from violence against FSW increased during the pandemic.

There are fewer studies examining the experiences and deaths of sexually exploited adolescent girls (SEAG). Sexual exploitation in this context is defined by the Convention on the Rights of the Child (<18 years) as “the exploitative use of children in prostitution or other unlawful sexual practices” or “the inducement or coercion of a child to engage in any unlawful sexual activity” [24]. The 2019 study on causes of FSW deaths in eight LMIC found that 13.4% of the 2,112 recorded deaths involved 11–19-year-olds and the leading causes of death in this age group were abortions (52%) followed by non-abortion maternal deaths (28%) [2]. While reports suggest social isolation, food insecurity, and economic precarity during the COVID-19 pandemic were associated with increased sexual violence and sexual exploitation of girls for food and money in Africa [25], there are no systematic studies quantifying the impact of the pandemic on the sexual exploitation of adolescent girls or the mortality of SEAG in LMIC.

The Community Knowledge Approach (CKA), validated in Bangladesh for capturing maternal and all-cause mortality, addresses gaps in mortality data by leveraging individuals’ unique knowledge of their own communities [26]. The CKA was also effectively applied in a 2019 multi-country study on FSW mortality [2]. In that context, “community” referred to FSWs residing in the same city who are familiar with one another. By partnering with trusted sex worker–led organizations (SWOs) and non-governmental organizations (NGOs), the CKA in 2019 enabled confidential reporting and identification of causes of FSW deaths.

The current study builds on previous work using the CKA to identify causes of death among FSWs. For this study, we collected data in the Democratic Republic of the Congo (DRC), Kenya, and Nigeria during the COVID-19 pandemic as these three countries reported the highest numbers of FSW deaths in the 2019 study, with a notable proportion attributed to maternal causes.

## Methods

### Study design

This study is part of a broader cross-sectional investigation into the health and nutrition of FSW and their children in the DRC, Kenya, and Nigeria. This component of the broader study leveraged the CKA methodology to identify causes of FSW deaths. FSW participants were asked to report on the deaths of FSW within their community of sex workers who they personally knew and about whose deaths they had first-hand knowledge.

### Study setting

Site selection for this study was based on the large number of deaths identified in the sub-Saharan countries of the DRC, Kenya, and Nigeria in the 2019 FSW mortality study [2]. Of note, the initial country selections for the 2019 study were based on the following five criteria: high country maternal mortality rate, estimated large number of FSW populations, high HIV infection rates among FSW, local partner organizations, and geographic regional diversity [2].

The current study was conducted in major urban centers in each of the three countries—Nairobi, Mombasa, and Kisumu (Kenya); Lagos, Calabar, and Abuja (Nigeria); and Bukavu and Kinshasa (DRC). These cities were accessible to the research team during the pandemic and have established FSW populations as well as local service-providing partner organizations that could facilitate community-based recruitment. Although the study design initially included three cities per country, data collection in Goma (DRC) was canceled due to inaccessibility in the setting of civil unrest and heightened security concerns.

### Study participants

Participants were female sex workers recruited by local SWOs in Kenya and Nigeria and by NGOs providing HIV services to FSW in the DRC. These organizations had previously collaborated on the 2019 FSW mortality study and are trusted within the FSW communities [2]. In Kenya, Nigeria, and Bukavu (DRC), convenience samples of 100 FSW were recruited per city from a variety of local venues including hot spots, bars, brothels, and streets. In the DRC, recruitment in Kinshasa was increased to 150 participants to compensate for the deficit resulting from the impasse into Goma.

FSWs was defined as adult females, aged 18 and older, “who sell consensual sexual services in return for cash or payment in kind, and who may sell sex formally or informally, regularly or occasionally” [27]. FSWs were eligible to participate in this study if they met the following inclusion criteria: (1) engaged in full-time sex work for the past three years, (2) sex work is the only source of income, (3) socially interactive with other FSWs, and (4) biological mother of at least one child aged five or younger. The latter criterion ensured adequate data for the maternal and child health component of the study.

To protect confidentiality of participants, no personally identifiable information was collected. Trained local partner staff in each country read and reviewed the study information sheet and the informed consent form in the local language with potential participants in coordination with the lead investigator. These documents included information about the sensitive nature (e.g., death reports of peers), purpose, and duration of the study, the potential harms and benefits of participation, details about compensation, and their rights as voluntary study participants to ask for clarifications, decline to answer questions, request breaks, withdraw from the study at any time, and seek mental health counseling through the local partner organizations. Following an interactive assessment of comprehension with ample opportunity for clarification and discussion, those who agreed to participate marked their consent with an “X” or checkmark to accommodate literacy limitations.

All participants were mothers of young children, and most brought children to the interview site. Caregivers were hired to allow mothers to fully engage in the interview. Participants and their children were provided with breakfast and lunch, and each participant also received cash compensation, based on local partner recommendations, ranging from $16 to $20 USD. These amounts were approved as appropriate and non-coercive by local ethics review boards.

### Data collection

In-person interviews were conducted using a structured paper-based questionnaire and verbally administered by the lead researcher (BW). Interviews took place in locations selected by the local partners to maximize participant safety and comfort. Interviews in Kenya and Nigeria were conducted in English by the lead researcher, with local staff present to clarify colloquial terms. In the DRC, questionnaires were translated into French and trained, bilingual staff members from local partner organizations translated the questions to Swahili in Bukavu and to Lingala in Kinshasa. Responses were translated to English and contemporarily recorded by the lead researcher.

Participants were first asked to report any known deaths of FSW, without any age restriction, in the current year, then in the previous year, continuing until no further deaths were recalled back to January 1, 2019, or the participant declined to continue. They were instructed to report only deaths they had personal knowledge of, excluding information they learned from other FSW or social media.

The following data were collected for each reported death: deceased’s name, age, date and city of death, participant’s relationship to the deceased, pregnancy status and gestational age if applicable, cause of death and other details of the death. If the death was abortion-related, detailed information was gathered on the method and location of death. For pregnancy-related deaths, further details included timing of death (during pregnancy, labor, or how long postpartum), type of care during childbirth, location and cause of death). Non-pregnancy-related deaths were categorized as: homicide, suicide, HIV/AIDS, unintentional injuries, suspected COVID-19, other cause, or unknown. Information about surviving children, their ages, and current caregivers was also recorded. For peripartum deaths, participants were asked whether the fetus was delivered, if it was live- or stillborn, and, if liveborn, whether the child was still alive; if not, the age at death was documented.

To prevent data duplication and minimize participant burden, any previously reported deaths were not re-recorded. After data collection in each city, the lead researcher and local staff reviewed all entries to identify and resolve duplicate reports based on name, age, and date of death. In addition to the de-duplication process performed in each city, this same researcher performed an extensive data quality assurance check at the conclusion of data collection in each country to ensure there were no duplicative deaths across the FSW community networks in different cities.

### Data analysis

Data on deaths were entered into Qualtrics XM/OS Platform by a contracted data entry specialist. A consultant, with access to all original questionnaires, conducted data cleaning. The cleaned data were then exported from Qualtrics to Excel for analysis. A spreadsheet was created to document all reported deaths that occurred during pregnancy, labor and delivery, or within 12 months postpartum. Two independent obstetrician-gynecologists, who were not involved in data collection, reviewed and coded each case using the International Classification of Diseases, 10th Revision (ICD-10). Each physician also classified the deaths as either direct or indirect maternal deaths. Discrepancies in coding were discussed between the reviewers until consensus was reached. Frequency tables were generated by cause of death, disaggregated by country, year, age, and location of death.

When asked to report on the deaths of their FSW peers, study participants reported the deaths of deceased females who were adolescents at the time of their deaths. Our reporting of these data is rooted in the widely accepted understanding that girls under the age of 18 are unable to consent to their own sexual exploitation and, therefore, are not engaged in consensual transactional sex despite perceived by FSW study participants as peers. Accordingly, our analyses differentiate between females who were 17 years and younger at the time of their death versus those who were 18 years and older. For reporting purposes, girls who were 17 years and younger at the time of their death are referred to as sexually exploited adolescent girls or SEAG.

### Ethics

The study was approved by the Institutional Review Board of Oregon Health and Sciences University, USA, (Protocol # STUDY00022962), and an ethics review board in each of the countries (Kenyatta University Center for Research and Safety, KU/ER/APPROVAL/VOL.1; Nigerian Institute of Medical Research IRb-22.009; Universite de Kinshasa Ecole De Sante Publique ESP/CE/093B/2023).

### Role of funder

There were two sources of funding for this study, one for data collection and one for data analysis and manuscript preparation. The funders had no role in the study design, data collection, data analysis, data interpretation, decision to publish, or manuscript preparation.

## Results

### Participants and dates of collection

The study included 250 FSW in the DRC, 301 in Kenya, and 302 in Nigeria, with approximately 100 participants in each of the eight cities except Kinshasa where there were 150 (Table 1). Data were collected in Kenya between February 10 and March 8, 2022; in Nigeria between March 22 and April 28, 2022; and in the DRC between January 27 and February 28, 2023. Each participant reported an average of 2.8 deaths in Kenya, 4.9 in Nigeria, and 5.9 in the DRC.

**Table 1:** Locations of interviews, dates of data collection, number of participants.

Country	City	Dates of data collection	No. Participants
Kenya	Nairobi	10 February 2022 - 18 February 2022	103
	Mombasa	22 February 2022 – 27 March 2022	99
	Kisumu	2 March 2022 – 8 March 2022	100
		***Total for Kenya***	***302***
Nigeria	Lagos	22 March 2022 – 31 March 2022	100
	Calabar	2 April 2022 – 14 April 2022	101
	Abuja	20 April 2022 – 28 April 2022	100
		***Total for Nigeria***	***301***
DRC	Bukavu	27 January 2023 – 5 February 2023	100
	Kinshasa	13 February 2023 –28 February 2023	150
		***Total for DRC***	***250***
**Total of all 3 countries**			**853**

### All causes of death

A total of 3,810 deaths of FSW were reported by 853 participants across the three countries (Table 2). Of these, 1482 deaths were reported in the DRC, 870 in Kenya, and 1458 in Nigeria.

**Table 2:** FSW and SEAG deaths by cause and country.

Country	Maternal Causes*			Homicide n (%)	HIV/AIDS n (%)	Unintentional Injury n (%)	Suicide** n (%)	COVID-19^ǂ^ n (%)	Other n (%)	Unknown n (%)	Total
**Abortion** n (%)	**Non-abortion** n (%)	**Total Maternal** n (%)
Kenya	108 (12.4)	30 (3.5)	138 (15.9)	254 (29.2)	181 (20.8)	56 (6.4)	69 (7.9)	20 (2.3)	83 (9.5)	69 (7.9)	870
Nigeria	340 (23.3)	188 (12.9)	528 (36.2)	306 (21.0)	126 (8.6)	94 (6.5)	151 (10.4)	2 (0.1)	124 (8.5)	127 (8.7)	1458
DRC	282 (19.0)	136 (9.2)	418 (28.2)	302 (20.4)	138 (9.3)	125 (8.4)	114 (7.7)	1 (0.1)	259 (17.5)	125 (8.4)	1482
Total	730 (19.2)	354 (9.3)	1084 (28.5)	862 (22.6)	445 (11.7)	275 (7.2)	334 (8.8)	23 (0.6)	466 (12.2)	321 (8.4)	3810

*Excludes maternal suicides; **Includes maternal suicides; ǂ Known or suspected

Across all three countries, maternal causes accounted for the largest percentage of deaths (28.5%). Homicide was the single leading cause (22.6%), followed by abortion (19.2%, representing 67.3% of maternal deaths). Other major causes included HIV/AIDS (11.7%), non-abortion maternal deaths (9.3%), maternal and non-maternal suicides (8.8%), and unintentional injuries (7.2%). Other causes, such as tuberculosis and malaria, accounted for 12.2% of deaths. Known or suspected COVID-19 caused 23 deaths (0.6%), with most reports coming from Kenya (n = 20). The cause of death was unknown for 321 cases (8.4%).

Maternal causes were the leading cause of death among FSW in the DRC (28.2%) and Nigeria (36.2%), while homicides were the leading cause in Kenya (29.2%). Homicide is among the top two causes of death among SEAG and FSW in all three countries.

In total, the majority of maternal deaths were due to abortion. When abortion-related deaths are considered separately from other maternal causes of death, abortion is the leading cause of death in Nigeria (23.3%), followed by homicide (21.0%) and then non-abortion maternal causes (12.9%). In the DRC, the three single leading causes were abortion (19.0%) followed by homicide (20.4%) and HIV/AIDS (9.3%), while in Kenya homicide (29.2%) was followed by HIV/AIDS (20.8%), then abortion (12.4%).

### Children left behind

The majority of deceased women (2,243; 58.9%) were mothers, leaving behind a total of 4,310 children – an average of 1.9 children per mother. Participants reported that care arrangements were made for nearly all (99.2%) of these children. Grandmothers were the most common caregiver (34.2%). Some orphaned children stayed with other FSW (10.2% of the caregivers) or were cared for by other family members including fathers, aunts, and older siblings, while the remaining children had no caregiver and were living on the streets starting as young as age three.

### Death by country, age group, and cause

Maternal causes were the leading cause of death in all age groups in the DRC and in all age groups except 36+ in Nigeria (Table 3). Maternal causes were also the leading cause of death for SEAG in all three countries.

**Table 3:** Causes of FSW and SEAG death by country and age group.

Country Age grp (%)	Maternal Deaths			Homicide n (%)	HIV/AIDS n (%)	Unintentional injuries n (%)	Suicide** n (%)	COVID-19^ǂ^ n (%)	Other n (%)	Unknown n (%)	Total N
**Abortion** n (%)	Non-abortion maternal* n (%)	Total Maternal Deaths* n (%)
***Kenya***											
11-17 (1.6) ^§^	3 (21.4)	1 (7.1)	4 (28.6)	3 (21.4)	2 (14.3)	1 (7.1)	0	0	0	4 (28.6)	14
18-25 (28.7)	50 (20.2)	7 (2.8)	57 (23.0)	86 (34.7)	38 (15.3)	16 (6.5)	19 (7.7)	4 (1.6)	15 (6.0)	13 (5.2)	248
26-35 (56.2)	46 (9.4)	19 (3.9)	65 (13.3)	141 (28.8)	114 (23.3)	30 (6.1)	39 (8.0)	11 (2.2)	49 (10.0)	40 (8.2)	489
36 + (13.4)	9 (7.6)	3 (2.5)	12 (10.1)	24 (20.2)	27 (22.7)	9 (7.6)	11 (9.2)	5 (4.2)	19 (16.0)	12 (10.0)	119
Total	108 (12.4)	30 (3.5)	138 (15.9)	254 (29.2)	181 (20.8)	56 (6.4)	69 (7.9)	20 (2.3)	83 (9.5)	69 (7.9)	870
***Nigeria***											
11-17 (5.6) ^§^	23 (28.0)	13 (15.9)	36 (43.9)	17 (20.7)	6 (7.3)	7 (8.5)	7 (8.5)	0	5 (6.1)	4 (4.9)	82
18-25 (54.3)	220 (27.8)	106 (13.4)	326 (41.2)	171 (21.6)	57 (7.2)	43 (5.4)	87 (11.9)	0	48 (6.1)	59 (7.5)	791
26-35 (32.4)	88 (18.6)	61 (12.9)	149 (31.4)	100 (21.1)	44 (9.3)	36 (7.6)	47 (9.9)	2 (0.4)	46 (9.7)	50 (10.5)	474
36 + (7.6)	9 (8.1)	8 (7.2)	17 (15.3)	18 (16.2)	19 (17.1)	8 (7.2)	10 (9.0)	0	25 (22.1)	14 (12.6)	111
Total	340 (23.3)	188 (12.9)	528 (36.2)	306 (21.0)	126 (8.6)	94 (6.5)	151 (10.4)	2 (0.1)	124 (8.5)	127 (8.7)	1458
***DRC***											
11-17 (15.4) ^§^	42 (18.4)	14 (6.1)	56 (24.6)	54 (23.7)	20 (8.8)	21 (9.2)	9 (3.9)	0	42 (18.4)	26 (11.4)	228
18-25 (56.9)	168 (19.9)	82 (9.7)	250 (29.6)	174 (20.6)	79 (9.4)	75 (8.9)	60 (7.1)	1 (0.1)	144 (17.1)	61 (7.2)	844
26-35 (25.0)	67 (18.1)	38 (10.3)	105 (28.4)	68 (18.4)	33 (8.9)	24 (6.5)	42 (11.4)	0	62 (16.8)	36 (9.7)	370
36 + (27.0)	5 (12.5)	2 (5.0)	7 (17.5)	6 (15.0)	6 (15.0)	5 (12.5)	3 (7.5)	0	11 (26.8)	2 (5.0)	40
Total	282 (19.0)	136 (9.2)	418 (28.2)	302 (20.4)	138 (9.3)	125 (8.4)	114 (7.7)	1 (0.1)	259 (17.5)	125 (8.4)	1482

*Excludes maternal suicides: 7 in Kenya, 31 in Nigeria, and 26 in the DRC.

**Maternal and non-maternal suicide

ǂ Known or suspected

§ Sexually exploited adolescent girls

When causes of death are disaggregated, abortion and homicides were the top two leading causes of death of FSW in nearly every age group in all three countries (Table 3). The exceptions were all deceased FSW 36 + years old, among whom HIV was the leading cause of death (tied with homicide in the DRC), and 26–35-year-olds in Kenya who were more likely to die from HIV than abortion. Non-abortion maternal deaths comprised the third leading cause of death in all age groups in Nigeria except those 36 + , accounting for 12.9% of all deaths in Nigeria.

Around one in five FSW deaths in Nigeria and the DRC, and nearly one in three deaths in Kenya, were due to homicide. The youngest homicide victim was 12 and the oldest was 59. The percentage of deaths due to HIV/AIDS tended to increase with the age of the deceased woman. Kenya had the largest number of FSW dying from HIV/AIDS and the highest percentage in all age groups. The youngest reported death from HIV was age 11 in the DRC and the oldest was age 50 in Nigeria. The highest percentage of deaths from suicide by age group was different for each country: in the DRC, it was 26–35-year-olds; in Kenya, it was 36 + , and 18–25 in Nigeria.

### Deaths of SEAG

Participants reported 324 deaths (8.6% of all deaths) among SEAG across all countries (Table 3). The proportion of SEAG deaths was highest in the DRC (15.4%), compared to 5.6% in Nigeria and 1.6% in Kenya. The youngest reported SEAG deaths occurred at age 11, one each in the DRC (HIV/AIDS) and Nigeria (IPV with miscarriage). Homicide and abortion were the top two causes of death among SEAG across all three countries. Homicide was the leading cause in the DRC and tied with abortion in Kenya, while abortion ranked first in Nigeria. There were six homicides of girls aged 12 and the youngest reported death from abortion was also aged 12, in the DRC. The third leading cause of death among SEAG varied by country: unintentional injuries in the DRC, HIV/AIDS in Kenya, and non-abortion maternal deaths in Nigeria.

Of the girls who died, 75 (23.9%) were reported to be HIV positive, with 52 (69.3%) not receiving antiretroviral therapy (ART) at the time of death. Over one-third of the girls (110; 35.0%) were pregnant when they died, and another third (105; 33.5%) were already mothers. The youngest maternal death occurred at age 11 due to a miscarriage after a physical assault, and the two youngest peripartum deaths, both aged 14, were reported in Nigeria.

In the DRC, participants were asked whether the deceased SEAG was the daughter of a FSW. Of the 228 SEAG deaths reported in the DRC, 68 (29.8%) were identified as daughters of FSWs.

### Maternal causes of death

Participants reported 1,148 maternal deaths (Table 4). There were 1,092 direct maternal deaths (95.1% of all maternal deaths; 28.7% of all deaths) and 56 indirect maternal deaths (4.9% of all maternal deaths). The leading cause of maternal death in all three countries was abortion: 63.5% of maternal deaths in the DRC; 74.5% in Kenya; and 60.8% in Nigeria. Hemorrhage and suicide were the second and third leading causes of maternal death overall (12.6% and 5.6%, respectively) and in all three countries. Notably, 10.0% of maternal death causes could not be specified.

**Table 4:** Causes of maternal deaths among FSW and SEAG.

Country	Direct Maternal								Indirect Maternal n (%)	TOTAL N
**Abortion** n (%)	**Hemorrhage** n (%)	**Obstructed Labor** n (%)	**Infection** n (%)	**Hypertensive disorder of pregnancy** n (%)	Suicide* n (%)	**Unspecified** n (%)	**Total Direct Maternal** n (%)
Kenya	108 (74.5)	10 (6.9)	2 (1.4)	0	2 (1.4)	7 (4.8)	3 (2.0)	132 (91.0)	13 (9.0)	145
Nigeria	340 (60.8)	74 (13.2)	17 (3.0)	2 (0.4)	1 (0.2)	31 (5.5)	68 (12.2)	533 (95.3)	26 (4.7)	559
DRC	282 (63.5)	61 (13.7)	6 (1.4)	3 (0.7)	5 (1.1)	26 (5.9)	44 (9.9)	427 (96.2)	17 (3.8)	444
Total	730 (63.4)	145 (12.6)	25 (2.2)	2 (0.4)	8 (0.7)	64 (5.6)	115 (10.0)	1092 (95.1)	56 (4.9)	1148

*During pregnancy and up to 12 months post-partum

### Deaths from abortion

Participants reported the method used to induce abortion in 700 (95.9%) of the deaths. Across all three countries, the most reported methods were pills and herbs. Pills were reported in 39.6% of abortion deaths. Among these, misoprostol was used in 53 cases, mifepristone in two, quinine or other non-specified pills in 50, and an unidentified pill in 140 cases (48.4% of all pill-related deaths). Pills accounted for 49.3% of abortion deaths in the DRC, 30.0% in Nigeria, and 44.4% in Kenya.

Herbs were used in 33.3% of cases, with the highest proportion in Nigeria (42.9%), followed by Kenya (33.3%) and the DRC (21.6%). Although names of specific herbs were generally not provided, they were either ingested as a “concoction” or inserted vaginally.

Surgical procedures caused 8.8% of abortion deaths—most commonly in Nigeria (11.2%), followed by the DRC (7.8%, with 19 of 22 cases in Kinshasa), and Kenya (3.7%).

In 8.6% of deaths, two or more methods were combined—e.g., a pill followed by an herb—or a second method was used after the failure of the first. The location of abortion-related deaths varied by country: in Nigeria, most abortion deaths occurred in brothels (30.7%); in the DRC, in small, informal hospitals (37.6%); and in Kenya, in the home (38.2%).

### Peripartum deaths

Hemorrhage was the top cause of peripartum deaths in all countries. Other causes included obstructed labor, infection, and hypertension. In the DRC, the majority of peripartum deaths were reported to occur in informal hospitals (55.4%), which do not provide skilled care, and 32.7% in hospitals (government, private, or NGO) (Table 5). In Nigeria, most peripartum deaths occurred in brothels (30.7%), with 23.5% in a government, private, or NGO hospital and 22.9% at homes of traditional birth attendants (TBA). Nearly all peripartum deaths reported in Kenya occurred in a government, private, or NGO hospital (82.6%). The age range for peripartum deaths was 14–42.

**Table 5:** Location of peripartum deaths among FSW and SEAG.

Country	Government, private, NGO hospitals n (%)	Informal hospital n (%)	Brothel n (%)	Home n (%)	TBA Home n (%)	Field, on the road, or other n (%)	En route to hospital n (%)	Total N
Kenya	19 (82.6)	0	0	3 (13.0)	0	0	1 (4.3)	23
Nigeria	36 (23.5)	9 (5.9)	47 (30.7)	17 (11.1)	35 (22.9)	5 (3.3)	5 (3.3)	153
DRC	33 (32.7)	56 (55.4)	2 (2.0)	2 (2.0)	0	8 (7.9)	1 (1.0)	101
Total	88 (31.5)	65 (23.3)	49 (17.6)	22 (7.9)	35 (12.5)	13 (4.7)	7 (2.5)	279

TBA- traditional birth attendant

### Birth outcomes for peripartum deaths

Of the 280 peripartum deaths, there were 50 stillbirths (17.9%), 47 neonatal deaths (16.8%) with 42 dying within seven days, 75 (26.6%) died in utero, and 105 infants (37.5%) who survived (Table 6). The percentage of infants who survived was similar across the three countries.

**Table 6:** Birth outcomes for peripartum deaths among FSW and SEAG.

Country	Fetal demise n (%)	Stillbirth n (%)	Neonatal death n (%)	Lived n (%)	Unknown n (%)	Total N
Kenya	1 (4.3)	6 (26.1)	8 (34.7)	8 (34.7)	0	23
Nigeria	46 (29.7)	28 (18.1)	22 (14.2)	56 (36.1)	3 (1.9)	155
DRC	28 (27.5)	16 (15.7)	17 (16.7)	41 (40.2)	0	102
Total	75 (26.8)	50 (17.8)	47 (16.8)	105 (37.5)	3 (1.1)	280

### Other deaths during pregnancy

Excluding peripartum deaths, participants reported 126 deaths of FSW during pregnancy. These deaths were due to homicide (54.0%), suicide (35.8%), unintentional injuries (11.9%), and unknown causes (9.7%). Reasons for suicide during pregnancy included lack of resources to care for a new baby, recent HIV diagnosis, and despondency over lack of support.

### Homicide

Homicide was the leading cause of death among FSW in Kenya and the DRC and the second leading cause in Nigeria, accounting for 862 deaths (22.6% of all reported deaths; see Table 2). Perpetrators were identified in all homicide cases and many included physical and sexual mutilation. Clients were the most commonly reported perpetrators (46.6%), followed by gang members (12.9%) and other FSW (11.5%). Additional perpetrators included long-term clients, police, and wives of clients. Participants provided details of the circumstances surrounding the homicides including reports of physical and sexual mutilation. Those committed by clients frequently followed disputes over payment, while other homicides occurred during police raids on brothels or when FSW were involved in looting food warehouses during the COVID-19 pandemic. Homicides of FSW by another FSW were reportedly due to conflicts related to a client.

### Suicide

A total of 334 maternal and non-maternal suicides were reported, representing 8.8% of all deaths. Among those who died by suicide, 28.7% were HIV-positive, including 11 who were pregnant at the time of death. The youngest suicide was reported at age 14 and the oldest at 47. Nearly one in five (19.2%) were pregnant or postpartum at the time of death. Contributing factors included food insecurity, recent HIV diagnoses, economic hardship—especially the inability to care for children—and conflicts with long-term clients. The most commonly reported methods were poisoning, particularly with *otapiepia* (a local insecticide used in Nigeria), and hanging.

### HIV/AIDS

HIV/AIDS accounted for 11.7% of all reported deaths. In 91.9% of cases, participants reported that the deceased had either never initiated treatment, often due to a recent diagnosis, or had defaulted. Lack of food was cited as the primary reason for treatment interruption (23.0%). Other contributing factors included limited access to antiretrovirals, substance use, depression, internalized stigma, and denial of diagnosis.

### Unintentional Injuries

There were 275 deaths due to unintentional injuries, with vehicular accidents comprising the majority (84.4%). Of these, motorcycles were involved in 40.0% of cases, cars in 31.9%, and buses or trucks in 28.0%. Many of these incidents occurred while women were walking to or from work. Other reported causes of deaths from unintentional injury included accidental electrocution and drowning.

### Other causes of death

There were 466 reported deaths from other causes (Table 7). Tuberculosis (TB) caused 4.7% of all deaths in the DRC. The age range for deaths from TB was 15–40 (mean 24 years old). Malaria, overdose, cancer, and other infectious diseases accounted for 0.7-3.4% of deaths in each country. Several causes were reported primarily in one country or city: of the 85 deaths from TB, 69 were reported in the DRC, with 59 of these in Kinshasa. All 35 deaths from Buruli ulcer were reported from the DRC with 34 reported in Kinshasa. And of 75 deaths from malaria, 40 were reported in the DRC. The majority of the deaths from cancer were reported in Kenya (30 of 36), of which nine were due to cervical cancer and reported in Kisumu. A small number of deaths were reported from malnutrition, fever, syphilis, and gonorrhea.

**Table 7:** Causes of death classified as “other” among FSW and SEAG.

Country	TB	Malaria	Overdose	Chronic diseases	Cancer	Buruli ulcer	Other infectious diseases	Other	Unknown	Total
n (% of all deaths ‘N’ in country)									
Kenya *(N = 870)*	5 (0.6)	7 (0.8)	7 (0.8)	17 (2.0)	30 (3.4)	0	9 (1.0)	7 (0.8)	1 (0.1)	83 (9.5)
Nigeria *(N = 1458)*	11 (0.8)	28 (1.9)	24 (1.6)	21 (1.4)	2 (0.1)	0	11 (0.8)	22 (1.5)	5 (0.3)	124 (8.5)
DRC *(N = 1482)*	69 (4.7)	40 (2.7)	25 (1.7)	7 (0.5)	4 (0.3)	35 (2.4)	31 (2.1)	45 (4.4)	3 (0.2)	259 (17.5)
Total *(N = 3810)*	85 (2.2)	75 (2.0)	56 (1.5)	45 (1.2)	36 (0.9)	35 (0.9)	51 (1.3)	74 (1.9)	9 (0.2)	466 (12.2)

## Discussion

This is the first known study to document mortality among FSW and SEAG during the COVID-19 pandemic. Across three sub-Saharan African countries, maternal mortality and homicide emerged as the leading causes of death. Maternal deaths—primarily due to abortion—were the leading cause in the DRC and Nigeria, while homicide was the leading cause in Kenya. Among SEAG, maternal mortality due to abortion was the leading cause in all three countries, followed by homicide. To our knowledge, this is also the largest and first in-depth analysis of mortality among SEAG, a critically understudied group.

Our findings align with earlier research identifying maternal causes as the leading contributor to deaths among FSW, and homicide as the second leading cause of death. Despite their vulnerability, mortality among SEAG and FSW remains largely undocumented, with the first major study only published in 2016 [28,29]. While pandemic-related mortality has been widely studied, including among vulnerable populations, these women and girls remain overlooked by mortality surveillance and studies.

### Maternal mortality

As in our study from 2019, maternal mortality is the leading cause of death among FSW [2]. While we could not calculate maternal mortality ratios, the mortality among SEAG and FSW is high compared to WHO data for each country [30]. This study also reconfirms that abortion represents 60–75% of all maternal deaths among SEAG and FSW in these countries.

In Kenya, 82.6% of peripartum deaths occurred in a government, private, or NGO hospital which suggests that FSW are accessing skilled attendants, but timing and quality of care may contribute to mortality. In the DRC and Nigeria, the majority of peripartum deaths occurred without skilled care, primarily in informal hospitals in the DRC, and in brothels, at home or TBA home in Nigeria. Access to skilled birth attendantse is a priority to prevent future deaths. The COVID-19 pandemic is known to have affected access to and utilization of maternal health services and may have been a factor in the high proportionate maternal mortality [13].

Abortion resulted in the majority of maternal deaths across all three countries. It was the leading cause of death in Nigeria and the second leading cause in the DRC. In Nigeria and Kenya, the largest percentage of deaths from abortion occurred in brothels and other locations outside of hospitals suggests barriers to quality post-abortion care. This may also reflect the economic impact of the pandemic on FSW who experience food insecurity and economic precarity at baseline. This situation continues to underscore the challenges for many poor FSW to access effective long acting and safe contraception and, where there is a complication from an abortion, to access quality post-abortion care.

### Homicide

Homicide was the leading cause of death in Kenya, the second leading cause in the DRC and Nigeria, and included 68 pregnant FSW and SEAG across all three countries. Homicides are not one of the top five leading causes of mortality for women aged 15–49 in any of these countries, however [31]. For example, there were 449 and 725 homicides of women reported across Kenya in 2020 and 2023, respectively [32]. In comparison, our study found 254 homicides between 2019 and the first quarter of 2022 in three Kenyan cities alone. In Nigeria, there were 7,350 homicides of women reported between 2019 and 2021 [32] with one study finding 205 due to IPV in that same time period [23]. In our study, we documented 306 homicides in just three Nigerian cities between 2019 and the first third of 2022. No published DRC data are available for comparison.

FSW homicides reported in this study correspond with documentation of increased violence against women and FSW during the pandemic [33,34]. The percent of deaths due to homicide reported in this study is substantially higher in the period of this study (2019–2022/23) compared to the 2019 study [2]: DRC 20.4% vs 3.5%; Kenya 29.2% vs 15.7%; and Nigeria 21.0% vs 7.0%. This does not necessarily mean there was an increase in the FSW homicide mortality rate. This change could be due to shifts in the other causes of mortality. Nevertheless, conditions in these three countries during the pandemic may have exacerbated pre-existing violence against FSW where non-fatal conflicts with clients before the pandemic, often over payment of fees, may have become more violent during the pandemic resulting in more lethal encounters. This study also documented that 11.5% of FSW were murdered by other FSW, often related to conflicts over clients. While workplace violence in other occupations is documented, to our knowledge this is the first documentation of workplace homicides of FSW by other FSW.

While this study does not focus on an analysis of the homicides, death reports detail the brutal nature of these deaths including the insertion of broken bottles into vaginal and rectal cavities and other forms of sexual mutilation *(data not shown; analysis in progress)*. Beyond the tragic death of each of these girls and women, many of whom were mothers, the immediate and long-term impact of these homicides on the other women who witnessed them or found their friends is not measured, nor is the impact on their children, who may have witnessed the murders or seen their deceased mothers.

### Deaths of SEAG

The deaths of SEAG, identified as FSW by participants in this study, highlights the significant risks to these girls. SEAG deaths included girls as young as 11-years-old and others who were pregnant or already mothers. In the DRC, participants reported that 68 of 228 (29.8%) SEAG who died were daughters of FSW. These findings highlight the urgent need to identify SEAG and provide them and their children with safe shelter, medical care, and psychological support.

The disproportionately large percentage of SEAG deaths occurred in the DRC (15.4% of all deaths) compared to Kenya (1.6%) and Nigeria (5.6%). In addition, 29.8% of the SEAG in the DRC were daughters of FSW. The reasons for more SEAG deaths, and more deaths of daughters of FSW, in the DRC than in the other two countries is unclear but requires additional research.

The leading causes of death among SEAG mirrored those of adult FSW: a combination of maternal causes, the majority of which were due to abortion and homicide. In Nigeria, nearly half of SEAG deaths (43.9%) were due to maternal causes with the majority (around two-thirds) due to abortion. As with adult FSW, many of the girls were infected with HIV (23.9%) but the majority (69.3%) were not on treatment. Clearly, urgent programming is needed to protect girls, especially daughters of FSW, from being sexually exploited and extricating those who are exploited and providing them with appropriate medical and psycho-social support [35].

As we have documented in a previous study, many daughters of FSW are sexually exploited [28]. The limited studies on trafficking of children in Africa focus on children who are transported, especially across borders, rather than sexual exploitation of vulnerable girls in their own communities [36]. Evidence-based policies and programs are urgently needed to protect daughters from sexual exploitation and assist those who are exploited. Decisive action is necessary to safeguard the physical and mental health of the daughters and their children, as well as their rights to live free from exploitation.

### Deaths related to COVID-19

In this study, conducted in all three countries during the pandemic, there were very few reported deaths directly attributable to COVID-19. This is not surprising considering that there were far fewer deaths due to COVID-19 infection in sub-Saharan Africa compared to other regions of the world [37]. Nevertheless, in this study the cause of 8.4% of deaths was unknown and it is possible that some of these unknown causes could have been due to COVID-19.

However, the impact of COVID-19 lockdowns and preventive measures may have had a substantial impact on the ability of FSW to make a living, pay for food, provide for their children, and access health services, among other potential effects [38]. Studies have documented the impact of the pandemic on access to maternal health, although none of these studies address barriers to maternal healthcare specifically for FSW during the pandemic [11–16,39]. The economic impact of the pandemic may have limited pregnant SEAG and FSW from accessing quality maternal health services including skilled birth attendants and emergency care.

Corroborating the recognized increased risk for sexual exploitation of girls due to food insecurity contributing to “transactional sex” for food [25,40], a study from Uganda during the pandemic found that the ability to provide for the basic needs of the family was protective to girls against gender-based violence [41]. The limited studies on the economic impact of the pandemic on FSW suggest that many could not provide for the basic needs of their children [38,42,43], which may have increased the risk of sexual exploitation among daughters.

### Tuberculosis and Buruli ulcer

There are numerous studies on TB among FSW but other than our 2019 study, few report of deaths of FSW from TB. This is also the first known study to report deaths of FSW from Buruli ulcer. That the majority of deaths from TB and Buruli ulcer were reported in Kinshasa likely indicates serious health problems among FSW in Kinshasa that are untreated, which contribute to these deaths. Based on the reports of these deaths, it is highly likely that there are many more untreated FSW with TB in Kinshasa who urgently require treatment and may also infect other women as well as their children.

### Impacts of FSW deaths on their children

The majority of women who died (58.9%) and one third of SEAG were mothers, leaving behind 4,310 children ranging in age from newborns to adolescents. Most participants were able to provide detailed information about these children including their ages, sex, and current caregiver. Grandmothers were most commonly identified as caregivers, followed by other FSW suggesting that many orphaned children may remain in brothels. Some siblings were separated and a few of the children were living on the street. Participants often reported that the caregiver, especially elderly grandmothers, could not adequately feed the children. FSW caring for orphans also reported similar struggles. Previous research has identified malnutrition as the leading cause of death among children of FSW [44] and the loss of a mother likely heightens this risk substantially.

In addition to the physical risks, the emotional and developmental impacts of a mother’s death on orphaned children are unstudied among children of FSW. First-hand accounts of these deaths indicate that some children may have witnessed their mothers’ traumatic deaths, including homicide, suicide, abortion, or childbirth complications, especially when these occurred in brothels where the children lived. These traumatic experiences can trigger a cascade of adverse childhood experiences among these children that increase long-term risks for poor health and psychosocial outcomes into adulthood [45].

### Social determinants of health and health equity

Socioeconomic risk factors such as access to healthcare and food are foundational to the health of SEAG and FSW. Unmet socioeconomic need pushes FSW into precarious occupational conditions as FSW prioritize feeding their children and having the means to afford basic healthcare.

The primary need of the majority of mothers in sex work is to feed their children every day. The focus of many health agencies on HIV prevention through condom distribution does not adequately address the socioeconomic risk factors which underpin HIV transmission risk and likewise, food insecurity. Efforts, while worthwhile, are too narrow in scope to change the structural conditions of FSW and their children.

Governments and foundations focused on improving the health and wellbeing of low-income, street-based FSW could prevent HIV transmission and other priority issues by moving upstream to address unmet health-related social needs (HRSN), which push women into sex work and in turn, increase risk for HIV, violence, unplanned pregnancies, suicide, and maternal deaths. Concerted efforts towards closing HRSN gaps can address the key issues contributing to death among FSW. Without adequate interventions, poor health outcomes are likely to persist.

### Recommendations and future research

From increased economic precarity, food insecurity, and GBV, to decreased access to contraception and maternal and non-maternal health care services, intersecting COVID-19-related disruptions and lockdowns had serious implications for marginalized populations. Consistent with the extant literature in the general population, the COVID-19 pandemic likely significantly exacerbated the challenges and inequities experienced by FSW and SEAG.

Access to affordable health care, including reproductive health and mental health care services, is a critical HRSN and component of mortality reduction and surveillance. In partnership with SWOs, NGOs, and philanthropic foundations, governments can allocate resources to comprehensive, skilled, and respectful maternal and non-maternal health care services tailored to the needs and constraints of FSW and SEAG. Evidence-based violence prevention programs and strategies must be funded and ideally target both the seller and buyer populations of sex work. As an especially vulnerable group, all future research on violence against women must specifically include a focus on the unique needs and circumstances of SEAG and FSW with the goal of identifying preventive and protective strategies effective in this population.

With respect to SEAG, robust enforcement of criminal statutes could act as a powerful deterrent against the sexual exploitation of a minor. Furthermore, governments can partner with SWOs and NGOs to extricate SEAG and their children from situations of sexual exploitation and provide them with comprehensive care and education. Primary prevention programs should prioritize the daughters of FSW as many deaths may be avoided with more resources dedicated to prevention of sexual exploitation of girls.

Study participants reported that FSW living with HIV died due to lacking sufficient food to continue their ART. Food insecurity was also identified as a contributing factor in homicide and suicide deaths. While more widespread food insecurity was a complication of the pandemic, previous research suggests a chronicity to this unmet need among FSW that predates the pandemic. Governments, NGOs, and philanthropic foundations could provide food relief assistance to these women and girls which may ultimately also reduce mortality among the children of FSW [44].

Finally, the current study serves as an urgent call to protect and preserve the lives and health of these women and their children in sub-Saharan Africa. Routinely excluded from formal mortality surveillance systems and civil registration systems, the death toll of FSW and SEAG remains largely invisible on the global stage and without action or accountability. In countries with high quality mortality data, the data show that total death counts decreased between 2022 and 2023, and the leading causes of death reverted to pre-COVID-19 causes [46]. Due to nonexistent mortality surveillance systems for FSW, a statistical analysis of pre- and post-pandemic causes of death is unfeasible. Pandemic conditions such as high inflation persist in the study countries [47,48] and leading causes of death among FSW and SEAG may not have reverted to pre-pandemic causes, however, surveillance infrastructure and research are needed for an accurate assessment. Near real-time tracking of death counts and cause-of-death trends in this hard-to-reach population may be feasible through the integration of community-based mortality surveillance systems.

### Limitations

This study has several limitations. First, the use of CKA—while valuable for capturing mortality among FSW, a population often missed in routine surveillance—has inherent constraints. It relies on peer-reported information subject to inherent limitations, including recall bias, particularly over a multi-year period, and the potential for differential recall of recent, highly salient, or traumatic death. In addition, cause of death classification is based on participant reports rather than medical records, which may result in misclassification of cause of death, especially for conditions with overlapping symptoms or where no formal care was received.

Reporting completeness and accuracy may also vary across sites depending on the strength of social networks, local stigma and participants’ knowledge of deaths within their community. As a result, some causes of deaths may be underreported, while others may be disproportionately represented. In addition, data collection was conducted with a limited number of FSW participants in a few urban areas in each country. The total number of deaths identified is therefore likely an undercount.

Second, because this study does not include a defined population denominator, it is not possible to calculate mortality rates or directly compare risk across settings or time periods. Observed differences in the proportion of deaths due to specific causes (e.g., homicide or maternal causes) between this and prior studies may reflect shifts in the distribution of causes rather than true changes in cause specific mortality.

Third, the study design did not include triangulation of death reports with civil death registries as this would have required authorization from government officials, which was beyond the scope of this study. In addition, while the study sought information about the location of the deaths, including hospitals, it was not designed to access medical records of health facilities to confirm the deaths or causes of death. Such access would have required the approval of and coordination with many hospitals during the pandemic, which was considered logistically unfeasible. Anecdotally, study participants and local SWO and NGO partners shared that many deceased women are hastily and informally buried by brothel owners to avoid police involvement, reputational damage, and loss of business; therefore, it is unclear the extent to which triangulation with civil death registries would be possible.

Finally, the study relied on a convenience sample of FSW recruited from a limited number of urban sites per country. As such, the findings may not be representative of the causes of FSW and SEAG deaths in other communities in the countries.

### Strengths

This study has several notable strengths. It is one of the few to document causes of death among FSW and SEAG in three sub-Saharan African countries during the COVID-19 pandemic, a period in which routine mortality data collection was disrupted. The CKA enabled rapid identification of deaths in this hard-to-reach population, who are often invisible in official reporting systems. Our expanded CKA questionnaire captured not only causes of death but also underlying contributing factors, such as lack of access to life-saving medical care. It also gathered information about what happened to the children of the deceased mothers, a largely undocumented issue.

The individual interview format provided a private, nonjudgmental setting and encouraged openness and detailed reporting. Participants reported multiple deaths and appeared willing to continue until they had exhausted their knowledge, suggesting a high level of engagement. The chronological recall approach—starting with recent deaths and moving backward—helped structure memory and ensure that current causes of death were captured. Asking participants to report all deaths, rather than deaths from specific causes, reduced bias and enabled identification of underreported causes, such as suicide or abortion.

In terms of data quality, all respondents had firsthand knowledge of the deaths they reported and provided highly specific accounts. The fact that 8.4% of deaths were classified as “unknown cause” suggests respondents did not speculate when unsure of the cause of death, strengthening confidence in reported data. All 3,810 reports of deaths were recorded by the lead researcher (BW), ensuring consistency in data collection in every city.

The use of proportionate mortality allowed for a broad understanding of the distribution of causes of death among FSW during a dynamic and under-studied period. While this approach cannot estimate risk, it effectively highlights dominant causes of death—such as maternal mortality and homicide—and can be a powerful tool for identifying public health priorities in data-scarce settings.

## Conclusion

This study, which was conducted during the COVID-19 pandemic, identified 3,810 deaths of SEAG and FSW in the DRC, Kenya, and Nigeria. The large number of deaths recorded in just a few cities over a short period is deeply concerning. Many of these deaths were preventable and occurred among mothers working to feed their children, who died from lack of basic medical care, violence, food, and despair. Life-saving interventions for many of these leading causes of death are known, effective, and inexpensive: skilled healthcare, food for FSW with HIV, mental health services, and reproductive health services. These services should be accessible and affordable for SEAG and FSW.

The pandemic resulted in excess deaths due to indirect causes, disproportionately affected vulnerable groups, and shifted the patterns of leading causes of death. It is likely that indirect impacts of the pandemic contributed to deaths among SEAG and FSW in these countries. Many of the deaths among SEAG and FSW are undocumented and unseen, contributing to a lack of awareness and funding to prevent them. A striking finding was the disproportionate number of deaths of SEAG in the DRC as compared to Kenya and Nigeria. Further research is warranted to understand the dynamics impacting SEAG within the cultural, political, legal, and economic context of the DRC and to identify effective preventative and protective measures.

Although the World Health Organization declared the pandemic over in May 2023, conditions for SEAG and FSW in these countries have not improved. The defunding of programs by USAID and PEPFAR, ongoing food insecurity, and continued armed conflict have worsened the vulnerability of SEAG and FSW. Governments, donors, foundations, and NGOs, must act urgently, in collaboration with sex worker organizations, to address these risks and prevent further loss of life.

## Supporting information

S1 Checklist Inclusivity in global research. (DOCX)

S1 Table Categorical definitions of causes of death. (DOCX)
