# Supplementary material for: Deaths of female sex workers and sexually exploited adolescent girls during the COVID-19 pandemic in Kenya, Nigeria, and the Democratic Republic of the Congo: Results of a cross-sectional study
Source: PLOS Glob Public Health. 2026 Aug 12;6(8):e0005781. doi: 10.1371/journal.pgph.0005781 (PMC13466021; doi:10.1371/journal.pgph.0005781)
Supplement: Supplementary file 1 [file pgph.0005781.s001.docx]

Inclusivity in global research

PLOS’ policy on inclusivity in global research aims to improve transparency in the reporting of research performed outside of researchers’ own country or community and ensures that PLOS publications reporting global research adhere to high standards for research ethics and authorship. Authors of relevant research articles may be asked to complete the questionnaire below, which outlines ethical, cultural, and scientific considerations specific to inclusivity in global research. This questionnaire may be requested when researchers have travelled to a different country to conduct research, if research uses samples collected in another country, research with Indigenous populations or their lands, or if research is on cultural artefacts. Researchers travelling to another country solely to use laboratory equipment will not normally be required to complete the questionnaire. However, the questionnaire can be requested at the journal’s discretion for any submission – if you have been requested to complete this questionnaire by the PLOS journal you submitted to, please do so.

Please complete the questionnaire below and include this as a Supporting Information file with your manuscript. Note that if your paper is accepted for publication, this checklist will be published with your article in the supporting information files. Please ensure that you reference the checklist in the main body of your manuscript. We suggest adding a subsection ‘Inclusivity in global research’ to your Methods section and adding the following sentence: “Additional information regarding the ethical, cultural, and scientific considerations specific to inclusivity in global research is included in the Supporting Information (SX Checklist)”

Reported on page number:

Institutional Review Board of Oregon Health and Sciences University, USA, (Protocol # STUDY00022962); 2. Kenyatta University Center for Research and Safety, KU/ER/APPROVAL/VOL.1; 3. Nigerian Institute of Medical Research IRb-22.009; 4. Universite de Kinshasa Ecole De Sante Publique ESP/CE/093B/2023).

The questions have been designed to be applicable to a wide range of study types, and there are subsections for both human subjects research and non-human subjects research. If any of the questions are not relevant to your research please mark them as “N/A” as appropriate.

**Ethical considerations, permits and authorship**

*This section is applicable to all research types.*

Provide details as to who granted permissions and/or consent for the study to take place in the Methods section of your manuscript. This should include the names of **all** ethics boards, governmental organizations, community leaders or other bodies that provided approval for the study. If individuals provided approval refer to these people by their role or title but do not list their name(s).

If there were any deviations from the study protocol after approval was obtained please provide details of these changes in the Methods section of your manuscript.
Did this study involve local collaborators that are residents of the country where the research was conducted or members of the community studied? If you do not have any authors from said communities, please provide an explanation for this below.

Yes. The following local organizations partnered with us: Local organizations that partnered with us, thereby approving the study in their community, include: Bar Hostess Empowerment and Support Programme (BHESP; Kenya); SWOP Ambassadors (Kenya); Coast Sex Workers Alliance (COSWA; Kenya); Kisumu Sex Workers Alliance (KISWA; Kenya); Partners For Health & Development in Africa (PHDA; Kenya); Nigeria Sex Workers Association (NSWA; Nigeria); Royal Women Health and Rights Initiative (RWHARI, Nigeria); Initiative for Young Women’s Health and Development (IY-WHAD, Nigeria); Action Humanitaire pour la Sante et le Developpement Communautaire (AHUSADEC; DRC); Cadre de Recuperation et d’Encadrement pour l’Epanouissement Integral des Jeunes (CREEIJ; DRC); and the Association pour le Soutien, l’Education, la Promotion de la Vie et des Initiatives Communautaires (ASEPROVIC; DRC).

Yes, one of the co-authors, Ms. Grace Kamau, is the  Regional Coordinator of the Africa Sex Workers Alliance (ASWA)

Reported on page number: Not applicable.

Everyone listed as an author should meet PLOS’ criteria for authorship and all individuals who meet these criteria should be included in the author byline, rather than the acknowledgements. For further information please see the journal’s Authorship Policy.

**Human subjects research (e.g. health research, medical research, cross-cultural psychology)**

Did you obtain written informed consent from a representative of the local community or region before the research took place? How did you establish who speaks for the community? Details of written informed consent obtained from study participants should be reported separately in the Methods section of your manuscript.

We obtained consent from representatives of every the local community in which we conducted this study through their review of the study protocol, questionniares, information sheets and informed consent form. There was no written consent from some government representative in the local community as this is not required by our IRB or our local partners. Rather, the representatives who provided consent were sex worker-led organizations in Kenya and Nigeria and NGOs in the DRC where there were no sex worker-led organizations. We have worked with all of these partners before and know they speak for the community through their interaction with the participants. We also established who speaks for the community through our colleague at the African Sex Workers Alliance who knows all the local leaders of sex worker communities in Kenya and Nigeria. In the DRC where we did not have a sex worker organization to partaner with, we worked through extremely well established NGOs that provide services to FSWs in Bukavu and Kinshasa.

How did members of the local community provide input on the aims of the research investigation, its methodology, and its anticipated outcome(s)? At the outset, we had a three day meeting with numerous sex worker organizations in Nairobi, Kenya, who reviewed the protocol and questionnaire and provided input on compensation and some terminology. In Nigeria and the DRC, we had meeting in each city with our local partner to review all components of study and they provide input on where to conduct interviews as well as other logistical issues, such as meals, and addressing other issues, e.g., sick children who accompanies their mothers to the interview.

When engaging with the local community, how did you ensure that the informed consent documents and other materials could be understood by local stakeholders? They were first reviewed by the sex worker orgqanizations in Nairobi. Then they were reviewed and approved by every local partner. The lead research has worked with every partner in the past and has experience discussing the informed consent documents with local partners. Every local partner reviewed the informed consent documents in detail and approved them.

Will the findings of the research be made available in an understandable format to stakeholders in the community where the study was conducted (e.g. via a presentation, summary report, copies of publications, etc.)? Please provide details of how this will be achieved.

Yes. We have shared our findings with the local community going back to the first study in which we identified deaths of FSW in Cambodia, which was conducted in 2013. The lead researcher returned to Cambodia in 2016, after the results had been published, and shared the results at a national conference on HIV that focused on HIV among FSW. After our 2019 study, the lead researcher returned to the DRC in March 2020 and shared our preliminary results at meetings in Kinshasas and Bukavu before meetings in Nigeria and Kenya were cancelled due to the pandemic. We then prepared a report for every partner. After our 2022/23 study (this study), we received a small grant for the Lead Researcher to present the preliminary results with local partners and stakeholders in Kenya, Nigeria, and the DRC. When this study is published, we will summary it and share with all of our local partners. This is one of the reasons that keeping the numbers (n) in the tables is critically important: the numbers matter to the local partners as they indicate the number of teens and women in their communities who died: it is not about “percentages” but each teen and woman who died.

**Non-human subjects research using specimens/ animals collected as part of the study, or those housed in archival collections. Examples include archaeology, paleontology, botany and zoology.**

Did the permission you obtained from a local authority to perform the study include an agreement on access to outputs and benefit sharing? This may include procedures to enable fair distribution of the benefits and resources arising from the research performed. Please include any details of Prior Informed Consent and Benefit Sharing Agreements obtained. These may be required by field-specific regulations, for example the Convention on Biological Diversity (CBD) and the associated Nagoya Protocol.

Not applicable to our study.

If the material used in your study was imported, please A) provide the year it was imported and B) indicate whether permits were obtained to import/export the materials used, C) provide details of any permits obtained. If this information is not available, please indicate this.

Not applicable to our study.

If you used archival specimens, please state how the material used in your study was acquired by the institute it is held in and provide details of any permits obtained for the original excavations/ sample collection. If this information is not available, please indicate this.

Not applicable to our study.

How was the potential cultural significance of the materials collected in your study to local communities considered in your research design? Were Indigenous peoples and/or local researchers and institutions involved with archaeological excavations / collection of specimens? If so, please provide a description of their involvement.

Not applicable to our study.

If your manuscript includes photographs of human remains please indicate whether authors obtained permission from descendants or affiliated cultural communities to do so.

Not applicable to our study.
