# Supplementary material for: Deaths of female sex workers and sexually exploited adolescent girls during the COVID-19 pandemic in Kenya, Nigeria, and the Democratic Republic of the Congo: Results of a cross-sectional study
Source: PLOS Glob Public Health. 2026 Aug 12;6(8):e0005781. doi: 10.1371/journal.pgph.0005781 (PMC13466021; doi:10.1371/journal.pgph.0005781)
Supplement: Supplementary file 2 [file pgph.0005781.s002.docx]

**S1 Table.** Categorical definitions of causes of death

| **Cause of Death** | **Definition** |
| --- | --- |
| ***Maternal Deaths*** | |
| Abortion | A death was classified as secondary to an abortion when the deceased was reported to have taken an action for the purpose of “the deliberate interruption of an ongoing pregnancy by medical or surgical means…” *(WHO, 2025).* |
| Direct and indirect maternal deaths not due to abortion | A maternal death is the death of a woman while pregnant or within the puerperium (within 42 days of termination of pregnancy), irrespective of the duration and the site of the pregnancy, from any cause related to or aggravated by the pregnancy or its management, but not from accidental or incidental causes. Maternal deaths are subdivided into two groups: (1) direct obstetric deaths: those resulting from obstetric complications of the pregnancy state (pregnancy, labor and the puerperium), from interventions, omissions, incorrect treatment, or from a chain of events resulting from any of the above; and (2) indirect obstetric deaths: those resulting from previous existing disease or disease that developed during pregnancy and which was not due to direct obstetric causes, but which was aggravated by physiologic effects of pregnancy *(WHO ICD-MM, 2012)*.  Suicides were classified as direct maternal deaths when they occurred temporal to pregnancy, childbirth, and the puerperium. When suicide occurs between 42 days and one year postpartum, these are coded as late maternal deaths from direct obstetric cause *(WHO ICD-MM, 2012)*. |
| ***Non-maternal Deaths*** | |
| Homicide | A death was classified as a homicide when the reported circumstances and details surrounding the death provided clear evidence of the intent by one or more persons to cause serious harm or death to another person as per the WHO definition that, “Homicide is the killing of a person by another with intent to cause death or serious injury, by any means. It excludes death due to legal intervention and operations of war” *(WHO, 2019).* |
| HIV/AIDS | A death due to HIV/AIDS is defined as a death resulting from an illness consistent with HIV disease, including opportunistic infections or AIDS-defining conditions, in a person living with HIV, where HIV infection is the underlying cause of death. This includes opportunistic infections (e.g., tuberculosis, pneumocystis pneumonia) and AIDS-defining illnesses, and excludes deaths unrelated to HIV (e.g., trauma, accidents), even if the person is HIV-positive *(WHO ICD-10, 2019)*. |
| Unintentional injury | A death was classified as secondary to an unintentional injury when the reported circumstances and details surrounding the death provided clear evidence of a preceding unintentional injury as per the WHO definition that “unintentional injuries are subdivided into road traffic injuries, poisoning, falls, fires, drowning, and other unintentional injuries. The latter category includes exposure to animate and inanimate mechanical forces (including firearms); exposure to electric current, radiation and extreme ambient temperature and pressure, and to forces of nature; and contact with heat and hot substances, and venomous plants and animals” *(WHO, 2006)*. |
| Suicide | A death was classified as secondary to an unintentional injury when the reported circumstances and details surrounding the death provided clear evidence of an intention to die preceding an action capable of causing death as per the WHO definition that “Suicide is a death resulting from an intentional act of self-harm, where there is evidence (explicit or implicit) that the person intended to die” *(WHO ICD-11, 2019)*. Non-maternal suicides are separate from suicides that occurred temporal to pregnancy and childbirth, and up to one year postpartum, which were classified as direct maternal deaths *(WHO ICD-MM, 2012)*. |
| Other | A death was classified as “Other” when the reported circumstance and details surrounding the death provided clear evidence of a cause of death not otherwise covered by the defined causes of death, including:  Tuberculosis deaths are classified in ICD-10 under codes **A15–A19** (active tuberculosis) and **B90** (sequelae of tuberculosis) listed by the WHO Mortality Database as codes specifically for tuberculosis causes of death *(WHO, 2026)*.  Cancer deaths were classified based on reports of the specific diagnosis and location of malignancy, e.g., breast or cervical cancer.    Buruli ulcer deaths were classified based on reports of the specific diagnosis and location of ulcerating wounds, e.g. *“mbasu”* in local language.  Drug overdose deaths were classified based on reports of the specific substance used preceding the fatal overdose and a lack of evidence of intentionality to cause fatal self-harm.  Deaths due to chronic diseases and infectious diseases, were classified based on reports of the specific diagnosis and corroborating information surrounding the death, e.g., asthma, cerebral malaria, typhoid, and yellow fever.  Death due to “other” causes were classified based on reports of the specific diagnosis and corroborating information surrounding the death, including advanced anemia, malnutrition, infected wound, and complications from surgery or a post-operative infection. |
| Unknown | A death was classified as “Unknown” when the information reported lacked specificity as to the cause of death and was insufficient to determine a clear cause of death, e.g. vomiting and diarrhea, as per ICD-10 code R99 (ill-defined, unspecified and unknown causes of death) *(WHO ICD-10, 2019)*. |

**Table References**

WHO (2025). Abortion Factsheet. 8 December 2025. <https://www.who.int/news-room/fact-sheets/detail/abortion>

WHO ICD-MM (2012). The WHO Application of ICD-10 to deaths during pregnancy, childbirth and the puerperium: ICD-MM. <https://iris.who.int/server/api/core/bitstreams/1bbeb2a8-e4c0-46c8-aa0d-0056627a17b5/content>

WHO (2019). Homicides. WHO Global Health Estimates. <https://apps.who.int/violence-info/homicide/>

WHO ICD-10 (2019). International statistical classification of diseases and related health problems (10th Revision). <https://icd.who.int/browse10/2019/en>

WHO (2006). Injury Prevention and Control. A guide to developing a multisectoral plan of action, surveillance, and research. <https://applications.emro.who.int/dsaf/dsa730.pdf>

WHO ICD-11 (2019). International classification of diseases for mortality and morbidity statistics (11th Revision). <https://icd.who.int/en/>

WHO (2026). Tuberculosis Factsheet. 24 March 2026. <https://www.who.int/news-room/fact-sheets/detail/tuberculosis>
